# First Documented Case of Percutaneous Endoscopic Gastrostomy (PEG) Tube-Associated Bacterial Peritonitis due to *Achromobacter* Species with Literature Review

**DOI:** 10.1155/2020/4397930

**Published:** 2020-01-16

**Authors:** Nishant Tripathi, Niki Koirala, Hirotaka Kato, Tushi Singh, Kishore Karri, Kshitij Thakur

**Affiliations:** ^1^Department of Internal Medicine, University of Kentucky, Lexington, KY, USA; ^2^Department of Clinical Pharmacy, University of Kentucky, Lexington, KY, USA

## Abstract

*Introduction. Achromobacter* species (spp.) peritonitis has seldom been identified in medical literature. Scarce cases of *Achromobacter* peritonitis described previously have been correlated with peritoneal dialysis and more sparingly with spontaneous bacterial peritonitis. *Achromobacter* exhibits intrinsic and acquired resistance, especially in chronic infections, to most antibiotics. This article conducts a literature review of all previously reported *Achromobacter* spp. peritonitis and describes the first reported case of *Achromobacter* peritonitis as a complication of percutaneous endoscopic gastrostomy (PEG) tube placement. *Discussion. Achromobacter* peritonitis as a complication of PEG-tube placement has not been previously reported. In our patients' case, the recently placed PEG-tube with ascitic fluid leakage was identified as the most plausible infection source. Although a rare bacterial peritonitis pathogen, *Achromobacter* may be associated with wide antimicrobial resistance and unfavorable outcomes. *Conclusion.* No current guidelines provide significant guidance on treatment of PEG-tube peritonitis regardless of microbial etiology. Infectious Disease Society of America identifies various broad-spectrum antibiotics targeting nosocomial intra-abdominal coverage; some of these antimicrobial selections (such as cefepime and metronidazole combination) may yet be inadequate for widely resistant *Achromobacter* spp. Recognizably, the common antibiotics utilized for spontaneous bacterial peritonitis, i.e., third generation cephalosporins and fluoroquinolones, to which *Achromobacter* is resistant and variably susceptible, respectively, would be extensively insufficient. Piperacillin/tazobactam (P/T) and carbapenem were identified to provide the most reliable coverage *in vitro*; clinically, 5 out of the 8 patients who received either P/T or a carbapenem, or both, eventually experienced clinical improvement.

## 1. Introduction

*Achromobacter* species (spp.) are nonfermenting, oxidase positive, catalase positive, aerobic, and motile Gram-negative rods [1]. As environmental organisms, *Achromobacter* spp. are naturally distributed in aqueous surroundings including soil and water [2–4]. Similarly, they may contaminate fluids in the health care setting, and have been considered infectious etiologies in nosocomial infection outbreaks associated with contaminated fluids [5], pressure transducers [6], incubators [7], and disinfectants [7]. Although opportunistic organisms [8], *Achromobacter* spp. are deemed to be the etiology for a myriad of infectious diseases with significant morbidity and mortality such as biliary tract sepsis [9], meningitis [10], pneumonia [11], spontaneous bacterial peritonitis [12, 13], peritoneal dialysis associated peritonitis [5, 14], urinary tract infection [11], conjunctivitis [15], osteomyelitis [16], prosthetic knee infection [17], mesh infection [18], necrotizing pancreatitis [19], prosthetic valve endocarditis [20], bacteremia [6, 11, 21], and cystic fibrosis [4, 22], among others. While the majority of these diseases occur as a polymicrobial infection in immunocompromised states such as cancer [21], human immunodeficiency virus/acquired immunodeficiency syndrome [23], chronic renal failure or diabetes mellitus [20], a portion of them occur due to nosocomial outbreaks in immunocompetent hosts [11, 24].

The organism is highly resistant to commonly utilized antibiotics in intra-abdominal infections. Piperacillin/tazobactam (P/T) and carbapenems show activity *in vitro* and are considered the most active antimicrobials against *Achromobacter* spp. [2]. On the other hand, sulfamethoxazole/trimethoprim may be a reasonable non beta-lactam alternative [25]. Due to the extensive inherent and acquired resistance to significant number of antimicrobials, providing dual coverage for chronic infections may be prudent [25].

## 2. Case Summary

A 65-year-old Caucasian male with a history of chronic alcoholism (without cirrhosis or ascites), hypertension and prior tonsillar adenocarcinoma status post chemo-radiation presented to a regional hospital with progressive fatigue and jaundice, epigastric pain, and decreased appetite causing twenty-five-pound weight loss spanning over a month. Leukocytosis along with lactic acidosis was observed. Patient was treated empirically with P/T for biliary pancreatitis secondary to choledocolithiasis. Initial endoscopic retrograde cholangiopancreatography (ERCP) on day (D) 2 of hospitalization identified distal common bile duct stone; sphincterotomy was performed with 7- French 5 cm plastic common bile duct stent placement. Despite the interventions, liver enzymes continued to rise; antibiotic was altered to levofloxacin and metronidazole on D4 to cover intra-abdominal infections and a repeat ERCP was performed. Recently placed CBD stent, which appeared clogged with debris, was removed uneventfully with snare. Appropriate drainage from CBD and cystic duct was confirmed; however, gall bladder could not be visualized with contrast. Due to the development of pneumonia, antibiotics were switched to linezolid and doxycycline on D6 of hospitalization. On D11, due to poor nutritional status and emerging aspiration pneumonia, PEG-tube was inserted. Subsequently, developing and rapidly worsening ascites and ascitic fluid leakage around the PEG-tube insertion site was identified. Paracentesis was performed on D13 of hospitalization without evidence of peritonitis; however, antibiotics were escalated to meropenem and linezolid due to worsening clinical status. Due to thrombocytopenia, linezolid was discontinued, and antimicrobial therapy was narrowed to meropenem on D17. Due to worsening of ascitic fluid leakage around the PEG insertion site, and exacerbation of liver enzymes, the patient was transferred to our academic facility for further management, including possible cholecystectomy on D17.

Upon transfer, the patient was hemodynamically stable with a constellation of physical exam findings significant for leaking ascites, icterus, spider angioma, fine crackles throughout lung fields, and 3+ bilateral pitting edema; no evidence of encephalopathy was noted. The laboratory tests were remarkable for white blood cells 21,600/mcl, serum creatinine 0.55 mg/dl, aspartate aminotransferase 201 U/L, alanine transferase 74 U/L, alkaline phosphatase 458 U/L, total bilirubin 9.0 mg/dl, serum albumin 1.8 g/dl, and INR 1.5. Patient was further diagnosed with alcoholic cirrhosis decompensated by ascites with MELD-Na score of 23, and suspected alcoholic hepatitis with Maddrey's discriminant function of 28. Antibiotic therapy was held until further work-up due to previously negative cultures and extended duration of therapy prior to transfer. Interventional radiology guided paracentesis was performed on D22 of hospitalization as bedside procedure was unsuccessful. Ascitic fluid analysis revealed albumin of 0.3 gm/dL and polymorphonuclear neutrophil count of 1950 cells/mcl. PEG-tube was retained to allow for maturation of PEG-tube tract; P/T and vancomycin were initiated and continued for two days (D22-D23); therapy was empirically altered to cefepime, metronidazole, and vancomycin to prevent acute kidney injury secondary to concomitant vancomycin and P/T use, which was continued for four days (D24-27). Ascitic fluid cultures revealed P/T susceptible and cefepime resistant *Achromobacter xylosoxidans* as the infectious etiology (Isolate was identified by MALDI TOF: MALDI Biotyper CA system. Please refer to Table 1 for minimal inhibitory concentration values). Empiric regimen of cefepime, metronidazole, and vancomycin was narrowed to P/T on D27. Bronchoalveolar Lavage on D27 due to worsening respiratory failure also identified *A. xylosoxidans*. Following five days of treatment with P/T, however, his abdomen demonstrated peritoneal signs with increasing ascites. Repeat paracentesis confirmed worsening bacterial peritonitis, with additional findings of vancomycin resistant *Enterococcus* and* Candida glabrata*. Despite broadened antimicrobial coverage with meropenem and vancomycin for four days (D28–D31), the patient deteriorated with acute kidney injury and hypotension, and was deceased within twenty-four hours of intensive care unit transfer.

## 3. Discussion

### 3.1. Nomenclature

The taxonomic designation for *Achromobacter* spp. has been inconsistent due to the limited differentiating capability of previous identification methods [26, 27]. Solely multilocus sequence typing (MLST) and species identification via *nrdA* sequence analysis for *Achromobacter* genus have been deemed to be effective in differentiating between various *Achromobacter *spp. [26, 27]. Prior to utilization of these methods, various alternate *Achromobacter* species might have been misidentified as *Achromobacter xylosoxidans*; currently, reclassification of *Achromobacter* species has resulted from utilization of these techniques [26–31]. The ascitic culture in our patient case was assessed by MALDI TOF MS (Matrix Assisted Laser Desorption/Ionization Time of Flight Mass Spectrometry); thus, exact species identification might not have been accurate. As such, our literature review attempts to include all previously documented *Achromobacter* species peritonitis cases to ensure an inclusive analysis.

### 3.2. Antimicrobial Susceptibility Patterns

*Achromobacter* susceptibility profile identifies the organism as being extensively resistant among various classes of antimicrobials, including fluoroquinolones, aminoglycosides, and the majority of broad-spectrum *β*-lactam antibiotics [32]. The resistance-nodulation-cell division (RND)-type multidrug efflux pumps, AxyABM [33, 34] and AxyXY-OprZ [35] have been associated with innate resistance. Essentially, resistant to all cephalosporins, except ceftazidime, due to the presence of AxyABM efflux system [29, 33], *Achromobacter* spp. may also contain AxyXY-OprZ efflux pump associated with aminoglycoside resistance [29, 35]. On the other hand, most isolates are typically susceptible to carbapenems, piperacillin, and P/T [29]. While a few acquired betalactamases have been described, overall acquired resistance mechanisms remain unknown [36–40].

### 3.3. PEG-Tube Associated Complications

This case report documents the first percutaneous endoscopic gastrostomy (PEG) tube-associated *Achromobacter* peritonitis. PEG-tube is a reliable source of enteral nutrition for patients with the inability to maintain oral nutrition on a long-term basis. Although minimally invasive, gastrostomy tubes have been associated with major complications such as peritonitis, major gastrointestinal bleeds, aspiration pneumonia, gastroenteric fistula, and gastrocolocutaneous fistula; minor complications include cellulitis, leakage around the tube, and granulation tissue at gastrostomy site [41–43]. In order to minimize these complications, the European Society for Clinical Nutrition and Metabolism guidelines on artificial enteral nutrition identifies a variety of contraindications to gastrostomy tubes including, serious coagulation disorders, INR greater than 1.5, platelets less than 50,000/mcl, marked peritoneal carcinomatosis, interposed organs (liver and colon), severe ascites, peritonitis, anorexia nervosa, severe psychosis, and discernibly limited life expectancy [44].

Specific risk factors for PEG-tube peritonitis include poor tissue healing, procedural, and technical issues, BMI over 30 kg/m2, albumin less than 2.5 g/dL, and dislodgement or reinsertion of tube [41, 42]. *Pseudomonas aeruginosa, Methicillin resistant Staphylococcus aureus*, and enteric Gram-negative rods are implicated as the most common infective etiologies of PEG site infections [45–47]. Abuksis et al. report 30-day mortality rate of 4.1–26% for stomal infections [48].

Despite the wide array of *Achromobacter* spp. infections previously reported, *Achromobacter* peritonitis is extremely rare and has been documented primarily in the setting of peritoneal dialysis. Despite the fact, *Achromobacter* infections in peritoneal dialysis are also exceptionally infrequent. Moreover, etiologies reported prior to *nrdA* sequencing might have been inaccurately identified as *A. xylosoxidans* and may rather represent *Achromobacter* spp. [11, 26–28]. In order to identify all reported and published cases of* Achromobacter* peritonitis, thorough PubMed (January 1^st^, 1966–April 7^th^, 2019) literature searches were conducted from March 20^th^, 2019 through April 7^th^, 2019, and October 10^th^, 2019 through October 21^st^, 2019 independently by both primary authors. Search terms that were used include “PEG or percutaneous endoscopic gastrostomy tube peritonitis” with no relevant results, “*Achromobacter* peritonitis” with four relevant results [49–52], and “*Alcaligenes* peritonitis” with additional 8 relevant results [12, 13, 53–58]. Five additional cases were identified through previously published case reports, and extensive search in PubMed with search terms “*Achromobacter xylosoxidans*”, “*Achromobacter”*, and “*Alcaligenes*” [5, 8, 14, 59, 60].

**Figure 1:**
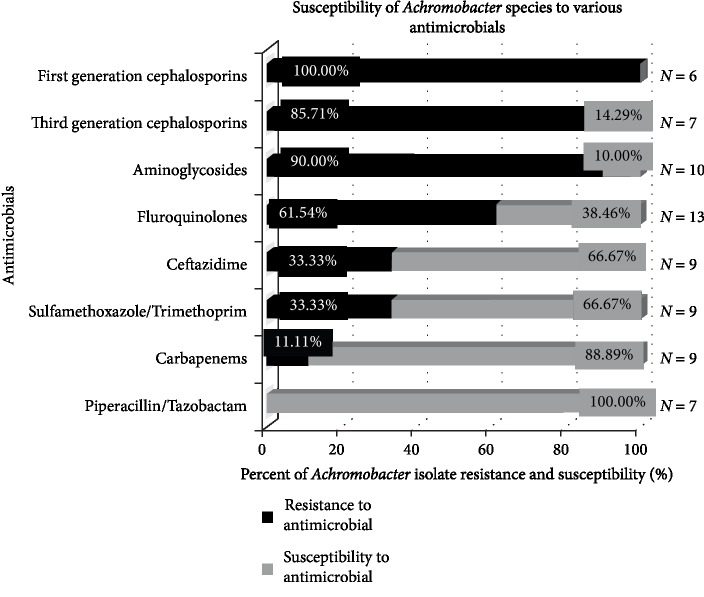
Susceptibility of *Achromobacter *spp. to various antimicrobials. Seven cases reported *Achromobacter* spp. sensitivity to piperacillin/tazobactam and all 7 isolates were susceptible to P/T. Similarly, 9 cases reported carbapenem sensitivity; 8 of 9 (88.89%) isolates were susceptible. SMZ/TMP and ceftazidime sensitivities were reported by 9 cases, of which 6 isolates (66.67%) were susceptible to each agent. On the other hand, 13 cases reported fluoroquinolones, of which, only 5 *Achromobacter* isolates (38.46%) were susceptible. Only 1 of 10 isolates (10%) were susceptible to aminoglycosides. None (0 out of 6) of the isolates were susceptible to first generation cephalosporins; and solely 1 out of 7 (14.29%) *Achromobacter* isolates were sensitive to third generation cephalosporins. Although *Achromobacter* susceptibility to other agents were also reported by most of the patient cases, sensitivity trends were not reported in this figure if data for at least five isolates could not be attained.

In totality, eighteen previous *Achromobacter* peritonitis cases were encountered; of which, two were diagnosed as spontaneous peritonitis [12, 13] while remaining sixteen cases of peritonitis were associated with peritoneal dialysis [5, 8, 14, 49–60]. No previous cases of PEG tube-associated *Achromobacter* peritonitis were discovered. Tables 1 and 2 summarize, in as much detail as could be obtained, the infectious diagnosis of each patient case, cultures, and sensitivities, antimicrobial therapies selected, and overall outcomes. Likewise, Figure 1 utilizes a bar graph to indicate the overall *in vitro* susceptibility profiles of the* Achromobacter* strains that were encountered in all nineteen cases of *Achromobacter* spp. peritonitis.

While 14 patients ultimately experienced clinical improvement, 4 patients were deceased. Patient responses to each antimicrobial group were assessed. P/T and carbapenem were identified to provide the most reliable coverage *in vitro*; clinically, 5 out of the 8 patients who received P/T or a carbapenem, or both, eventually experienced clinical improvement. Of the three patients who did not, patient in case nine was administered P/T but patient was deceased very shortly after presentation. Patient likely presented with high disease severity; thus, overall outcome may not reflect utility of the treatment. Likewise, patient in case thirteen was initiated on amikacin upon presentation, which was continued for seven days without confirmed susceptibility; the case report identified susceptible agents, which did not include any aminoglycosides. Following seven days of likely inadequate treatment, therapy was escalated to imipenem and P/T dual-therapy, which was narrowed to imipenem. Despite the eventual initiation of antimicrobials with verified susceptibility, patient failed to respond to therapy despite over 30 days of carbapenem therapy. Such response may have been due to delayed removal of peritoneal dialysis catheter as well as delayed initiation of adequate antimicrobial coverage. Finally, patient described in this case report was initiated on P/T and vancomycin; empirically, therapy was altered to cefepime, metronidazole and vancomycin for four days to prevent acute kidney injury. Unfortunately, the ascitic fluid culture resulted cefepime-resistant but P/T sensitive *Achromobacter* spp. Despite definitive treatment with P/T for five days and meropenem for four additional days, no recovery was observed. Such outcome may have resulted due to interruption of appropriate treatment as well as retention of PEG-tube despite multiple positive ascitic fluid cultures. No trend in antibiotic selection was observed among nine additional patients with clinical recovery who received agents other than carbapenems and P/T. While the International Society for Peritoneal Dialysis (ISPD) guideline suggests structurally similar *Pseudomonas* associated peritonitis treatment with dual antibiotic regimen, the guideline makes no specific recommendations for *Achromobacter* treatment [43]. Per *in vitro* and in vivo data collected in this article and through numerous studies, P/T and carbapenems yield the most preferable clinical outcomes in *Achromobacter* peritonitis [2, 24, 25, 34].

PD catheter was removed in 9 out of 14 non-SBP patients with clinical cure. All patients with resolved infection received at least seven days of antimicrobial treatment. Five patients received seven to ten days; four patients received fourteen days; two patients received twenty-one days, and one patient received over 30 days of antibiotics. Glucose containing peritoneal dialysate and aqueous environment foster *Achromobacter* proliferation and associated infection. Moreover, *Achromobacter* colonies may have the ability to surround the PD catheter with biofilm [14, 52, 61, 62]. Bacteria in biofilms in vivo are significantly less susceptible to antimicrobials identified to have adequate coverage through laboratory testing *in vitro* [43]. This fact may further clarify the treatment failures, relapses, and patient deaths resulting in patients who received P/T or carbapenems in these case reports. In addition to appropriate antimicrobial therapy, case seventeen also highlights the importance of timely removal of peritoneal dialysis catheter. Donderski et al. [52] report multiple peritonitis relapse associated readmissions; fortunately, no further episodes were reported following PD catheter removal and a course of adequate antimicrobial regimen. Thus, multiple case studies highlight the importance of timely removal of *Achromobacter* infected PD catheters; Donderski et al. [52] identify catheter removal as the most preferred treatment method. ISPD Peritonitis Recommendations: 2016 Update on Prevention and Treatment identifies peritonitis as a common and serious complication of PD [43]. Severe or prolonged peritonitis can induce functional and structural deformations resulting in membrane failure. Gram-negative peritonitis presents higher risks of catheter loss and death compared to Gram-positive infections [63–67]. ISPD guideline recommends removal of PD catheter in cases of recurrent, relapsed, and refractory (lack of clearance of ascitic fluid despite 5 days of appropriate antimicrobial treatment) peritonitis in order to protect the peritoneal membrane [43]. Timely catheter removal is essential in recurrent episodes. Nonetheless, PD catheter or PEG-tube removal within ten days to four weeks of insertion may not allow respective insertion tracts to mature causing further leakage and complications [44, 68–70].

Additionally, ISPD suggests at least 3 weeks of treatment in nonpseudomonas Gram-negative, and polymicrobial PD peritonitis [43]. No current guidelines provide significant direction on treatment of PEG tube peritonitis regardless of microbial etiology. Nevertheless, clinical practice guidelines for antimicrobial prophylaxis in surgery strongly recommends prophylactic antibiotic use to significantly reduce the risk of peristomal wound infection associated with PEG-tube insertion [71]. ASPEN guidelines recommend gastropexy using temporary sutures or T-fasteners to secure the stomach to the abdominal wall [72]. These techniques allow for appropriate attachment of tube, minimizing potential for peritoneal leakage, and reducing difficulty and challenges associated with tube replacement, if indicated [72]. Upon development of peritonitis, tube feeds should be held with initiation of broad-spectrum antibiotics. Evidence of perforation should be assessed via abdominal imaging; potential for wound should be evaluated. Surgical consult to perform laparotomy should be obtained. As misplaced tube may cause peritonitis and death, test must be performed to detect misplacement of tube at exchange. In instances of early inadvertent gastrostomy tube removal, parenteral broad-spectrum antibiotics and monitoring for peritonitis signs are indicated [72]. Although recommendations for total duration of antibiotics for treatment of PEG-tube peritonitis could not be encountered in current literature, in case of suspected peritonitis, clinical knowledge extrapolated from similar gastrointestinal perforations suggests that antibiotics may be discontinued after seven days if peritonitis is not observed [70]. Longer duration of treatment may be required in cases of confirmed PEG-tube peritonitis, particularly in cases without adequate source control [70]. Due to the lack of data to guide treatment of PEG tube-associated *Achromobacter* peritonitis, we attempted to draw similarities between *Achromobacter* peritonitis due to PEG-tube and PD. Nonetheless, the authors acknowledge that one of the major differences between the two insertions is the accessibility of the peritoneal cavity; while PD catheter readily allows for diagnosis as well as intraperitoneal treatment of peritonitis, PEG tubes should ideally not provide direct access to the peritoneal cavity.

Multiple risk factors for PEG-tube related complications could be identified in the patient presented in our case study. Undiagnosed advanced liver disease, severe ascites, hypoalbuminemia, prolonged hospitalization, and immunocompromise secondary to liver cirrhosis, all place the patient is at high risk for PEG-tube related complications. Polymicrobial nature of the patient's peritonitis also portends adverse outcomes; likewise, patient's ultimate demise indicates the severity of the disease.

## 4. Conclusion

*Achromobacter* spp. peritonitis as a complication of PEG-tube placement has not been previously reported. In our patients' case, the recently placed PEG-tube with ascitic fluid leakage was identified as the most plausible infection source. Although a rare bacterial peritonitis pathogen,* Achromobacter* spp. may be associated with wide antimicrobial resistance and unfavorable outcomes. Thus, clinicians should consider broad empirical antimicrobial regimen, with *Achromobacter* coverage, such as P/T or a carbapenem, if any concern for *Achromobacter* spp. secondary peritonitis associated with PEG placement exists, as they may not always represent clinical sample contamination. While the management of *secondary* peritonitis involving biliary stent and PEG-tube would generally include surgical intervention for infection source control with broad spectrum antibiotics targeting nosocomial coverage per Infectious Disease Society of America, some of antimicrobial selections (such as cefepime and metronidazole combination) may yet be inadequate for widely resistant *Achromobacter* species. Recognizably, the common antibiotics for *spontaneous* bacterial peritonitis, i.e., third generation cephalosporins and fluoroquinolones, to which *Achromobacter* is resistant and variably susceptible, respectively, would be extensively insufficient. Moreover, repeat ascitic fluid culture may be beneficial in verifying continued susceptibility of selected definitive antibiotics. In addition, as these pathogens are generally identified in polymicrobial infections, their presence may serve as a surrogate marker of unfavorable outcomes. Due to the high disease severity, delay or interruption in efficacious therapy may lead to increased mortality rates.

## Figures and Tables

**Table 1:** All reported cases of *Achromobacter* spp. peritonitis-demographics, coinfection etiologies, and catheter or tube removal status.

Case	Year	Age/sex	PD catheter or PEG-tube removal	Coinfection microbes
Bacterial peritonitis secondary to peritoneal dialysis				

1 [8]	1980	53/M	No	*Staphylococcus epidermidis*
2 [5]	1984	40/M	No	*Stenotrophomonas maltophilia*
3 [53]	1986	34/F	No	None
4 [54, 55]	1995	45/M	Yes. Day 48	None
5 [55]	1998	52/F	Yes. Day 10	None
7 [56]	Sep 2001	46/F	Yes. Day 6	None
8 [56]	Sep 2001	35/F	Yes. Day 8	None
10 [49]	2004	16/M	Yes. Day NR	None
11 [57]	2007	72/F	No	None
12 [58]	2010	51/F	Yes. Day 1	None
13 [59]	2011	74/F	Yes. Day 19	*Pseudomonas aeruginosa*
14 [60]	May 2012	82/F	No	*Achromobacter denitrificans*
15 [50]	Jul 2012	31/M	No	None
16 [51]	Jan 2014	60/F	No	None
17 [14]	2017	45/F	Removed prior to *Achromobacter* peritonitis	None
18 [52]	2018	27/F	Yes. Day NR. During third episode	None

Sponteneous bacterial peritonitis				

6 [12]	2000	43/M	N/A	None
9 [13]	2001	54/M	N/A	Likely *Escherichia coli*

Percutaneous endoscopic gastrostomy tube-associated peritonitis				

19	2018	65/M	No	*Enterococcus faecium*
				*Candida glabrata*

In case 7, PD catheter was replaced one-month postantibiotics. In case 8, catheter was replaced 6 weeks postantibiotics; PD catheter failed; subsequently, hemodialysis was initiated. Cases 11, 14, and 15 were reported as exit site infections. Case 16 was reported to be a tunnel infection. Abbreviations: PD = peritoneal dialysis, PEG = percutaneous endoscopic gastrostomy, NR = not reported, N/A = not applicable.

**Table 2:** All reported cases of *Achromobacter* spp. peritonitis-bacterial sensitivities, antibiotics selection with duration, and outcomes.

Case	Antimicrobial sensitivities	Antibiotics utilized	Antibiotic duration	Outcomes
Bacterial peritonitis secondary to peritoneal dialysis				

1 [8]	S: ampicillin, carbenicillin, colistin, smz/tmp	Carbenicillin IV	NR	Cure
	R: AG, tetracycline			
	I: cephalothin			

2 [5]	S: colistin, moxalactam, cefamandole	Moxalactam ^∗^	Likely 15 days	Deceased
	R: piperacillin, azlocillin, ceftazidime, cefoperazone, novobiocin, minocycline	Noxytiolin IP		
	I: rifampin, rosoxacin			

3 [53]	S: smz/tmp, AG, carbenicillin	Tobramycin IP	3 days	Relapse
		Cephalothin IP		
	R: ampicillin, cefamandole, cefoxitin, cephalothin, tetracycline, chloramphenicol	smz/tmp IP	10 days	Cure

4 [54, 55]	R: AG	Vancomycin IP	1 dose	Deterioration
		Gentamicin IP		
	*Complete sensitivity profile was not available. Initial response to gentamicin was documented by El-Shahawy, et al. [46].*	Gentamicin IP	NR	Deterioration
		Ciprofloxacin PO		
		Piperacillin IV	7 days	Cure

5 [55]	S: ofloxacin	Vancomycin IP	3 days	Cure
		Ceftazidime IV		
	R: AG, aztreonam, cefazolin, cefotaxime, cefoxitin, ceftriaxone, ceftazidime, cefuroxime, ciprofloxacin, imipenem, piperacillin, smz/tmp	Ceftazidime IV	14 days	
		Ofloxacin PO		

7 [56]	S: P/T	Cefazolin IP	3 days	Cure
		Tobramycin IP		
	R: ampicillin, cephalothin, ceftriaxone, AG, ciprofloxacin, smz/tmp	Amikacin IP	3 days	
		Ceftazidime IP		
		P/T IV	21 days	

8 [56]	S: piperacillin, ticarcillin, ceftriaxone, ceftazidime, smz/tmp	Cefazolin IP	3 days	Cure
		Tobramycin IP		
	R: AG	Amikacin IP	5 days	
		Ceftazidime IP		
		P/T IV	21 days	

10 [49]	S: ciprofloxacin, imipenem	Vancomycin IP	NR	Cure
		Ceftazidime IP		
		Ciprofloxacin PO		
	R: ceftazidime, smz/tmp	Ceftazidime IP		
		Amikacin IP		
		smz/tmp PO		
		Imipenem IP		
		Ciprofloxacin PO		

11 [57]	S: imipenem, P/T	Ceftazidime IP	1 dose	Cure
		Cefazolin IP		
	R: cefotaxime, AG	Ciprofloxacin PO	NR	
		Imipenem IV	3 doses	
	I: ciprofloxacin	Imipenem IP	30 days	
		Ciprofloxacin PO	10 days	

12 [58]	*Complete sensitivity profile was not reported but ampicillin/sulbactam, ciprofloxacin and cefepime were definitive therapies*	Ceftazidime IV	NR	Cure
		Cefazolin IV		
		amp/sulb IV	7 days	
		Ciprofloxacin^∗^	2 days	
		Cefepime^∗^	7 days	

13 [59]	S: ceftazidime, imipenem, meropenem, levofloxacin, piperacillin	Vancomycin IP	7 days	Deterioration
		Amikacin IP		
	R: NR	Imipenem/cil IP	7 days	
		P/T IP		
		Ceftazidime	NR	Deceased
		Cefazolin		
		Imipenem/cil		
		Vancomycin		
		Imipenem/cil IP	NR	
		Vancomycin IP		
		Imipenem/cil IV	29 days	
		Vancomycin IV		

14 [60]	S: ceftazidime, cefepime, sulfonamide, quinolones, carbapenem, P/T	Ciprofloxacin PO	14 days	Cure
	R: NR			

15 [50]	*Unable to obtain due to lack of article access*	Cefazolin IP	3 days	Cure
		Ceftazidime IP		
		Ceftazidime IP	4 days	

16 [43]	S: ciprofloxacin, others NR	Ciprofloxacin^∗^	NR	Cure
	R: NR			

17 [14]	S: amp/sulb, smz/tmp, carbapenem, cephoperazone/sulbactam, ceftazidime, P/T, tigecycline	Cephalosporin PO	14 days	Relapse
	R: 1st generation cephalosporin, ciprofloxacin	Cefazolin IP	7 days	Cure
		Ceftazidime IP		
		amp/sulb PO	14 days	

18 [52]	S: P/T, ceftazidime, imipenem	Ceftazidime IP	NR	Replase
		Cefazolin IP		
	R: ciprofloxacin, cefepime	Imipenem/cil^∗^	14 days	
		Imipenem IV	14 days	Replase
		Ceftazidime IV		
		Imipenem IV	14 days	Cure
		Ceftazidime IV		

Sponteneous bacterial peritonitis				

6 [12]	S: amox/clav, ceftazidime, P/T, imipenem, meropenem, cotrimoxazole	Ceftriaxone IV	10 days	Cure
	I: cefotaxime, ceftriaxone, ofloxacin, ciprofloxacin			
	R: AG, aztreonam, cefazolin, cefuroxime			
9 [13]	S: NR	P/T IV	Few hours	Deceased (few hours later)
	R: cefotaxime, aztreonam, AG, ciprofloxacin			

Percutaneous endoscopic gastrostomy tube-associated peritonitis				

19	S: P/T (≤2/4), smz/tmp (≤0.5/9.5), meropenem (0.25)	P/T IV	4 days	Deterioration
	R: aztreonam (16), cefepime (16), gentamicin (8)	Levofloxacin IV	2 days	Deterioration
		Metronidazole IV		
	I: amikacin (32), levofloxacin (4), tobramycin (8)	Linezolid IV	7 days	Deterioration
		Doxycycline IV		
		Meropenem IV	4 days	Adverse reaction
		Linezolid IV		
		Meropenem IV	2 days	Transferred to UK
		P/T IV	2 days	Adverse reaction
		Vancomycin IV		
		Cefepime IV	4 days	Deterioration
		Vancomycin IV		
		Metronidazole IV		
		P/T IV	5 days	Deterioration
		Meropenem IV	4 days	Deceased
		Vancomycin IV		

In case 2, the duration of treatment was not clearly reported but was likely 15 days; route of moxalactam was unreported. Case 12 did not report route of ciprofloxacin and cefepime; total of 20 days of antibiotics were administered as patient had presented with 5^th^ episode of peritonitis; subsequently, hemodialysis was initiated. Case 13 reported total of 48 days of antimicrobial treatment. Case 16 did not report full culture and sensitivities, route of ciprofloxacin and total duration of the treatment. Case 18 does not report route of imipenem/cilastatin. Abbreviations: S = Sensitive, R = resistant, I = intermediate, smz/tmp = sulfamethoxazole/ trimethoprim, P/T = piperacillin/tazobactam, amp/sulb = ampicillin/sulbactam, amox/clav = amoxicillin/clavulanate, cil = cilastatin, AG = aminoglycosides, IV = intravenous, IP = intraperitoneal, PO = oral, NR = not reported.

## References

[1] Yabuuchi E., Oyama A. (1971). *Achromobacter xylosoxidans* n. sp. from human ear discharge. *Japanese Journal of Microbiology*.

[2] Waters V. (2012). New treatments for emerging cystic fibrosis pathogens other than *Pseudomonas*. *Current Pharmaceutical Design*.

[3] Amoureux L., Bador J., Fardeheb S. (2013). Detection of *Achromobacter xylosoxidans* in hospital, domestic, and outdoor environmental samples and comparison with human clinical isolates. *Applied Environmental Microbiology*.

[4] Lambiase A., Catania M. R., Del Pezzo M. (2011). *Achromobacter xylosoxidans* respiratory tract infection in cystic fibrosis patients. *European Journal of Clinical Microbiology & Infectious Diseases*.

[5] Reverdy M. E., Freney J., Fleurette J. (1984). Nosocomial colonization and infection by *Achromobacter xylosoxidans*. *Journal of Clinical Microbiology*.

[6] Gómez-Cerezo J., Suárez I., Ríos J. J. (2003). *Achromobacter xylosoxidans* bacteremia: a 10-year analysis of 54 cases. *European Journal of Clinical Microbiology and Infectious Diseases*.

[7] Molina-Cabrillana J., Santana-Reyes C., González-García A., Bordes-Benítez A., Horcajada I. (2007). Outbreak of *Achromobacter xylosoxidans* pseudobacteremia in a neonatal care unit related to contaminated chlorhexidine solution. *European Journal of Clinical Microbiology & Infectious Diseases*.

[8] Igra-Siegman Y., Chmel H., Cobbs C. (1980). Clinical and laboratory characteristics of *Achromobacter xylosoxidans* infection. *Journal of Clinical Microbiology*.

[9] Teng S. O., Ou T. Y., Hsieh Y. C., Lee W. C., Lin Y. C., Lee W. S. (2009). Complicated intra-abdominal infection caused by extended drug-resistant *Achromobacter xylosoxidans*. *Journal of Microbiology, Immunology and Infection*.

[10] Bellissimo F., Pinzone M. R., Tosto S., Nunnari G., Cacopardo B. (2014). *Achromobacter xylosoxidans* meningitis in an immunosuppressed patient. *An International Journal of Medicine*.

[11] Marion-Sanchez K., Pailla K., Olive C., Le Coutour X., Derancourt C. (2019). *Achromobacter* spp. healthcare associated infections in the French West Indies: a longitudinal study from 2006 to 2016. *BMC Infectious Diseases*.

[12] Palmero Palmero C., Giráldez Gallego A., García Morillo S., Miranda Guisado M. L. (2000). Spontaneous bacterial peritonitis caused by *Alcaligenes xylosoxidans*. *Medicina Clinica*.

[13] Castellote J., Tremosa G., Ben S. L., Vazguez S. (2001). Spontaneous bacterial peritonitis due to *Alcaligenes xylosoxidans*. *The American Journal of Gastroenterology*.

[14] Tsai J. L., Tsai S. F. (2017). Case report: the first case of *Achromobacter xylosoxidans*-related tunnel infection in a patient receiving peritoneal dialysis. *Medicine (Baltimore)*.

[15] Arshad J. I., Saud A., White D. E., Afshari N. A., Sayegh R. R. (2019). Chronic conjunctivitis from a retained contact lens. *Eye Contact Lens*.

[16] Shinha T., Oguagha I. C. (2015). Osteomyelitis caused by *Achromobacter xylosoxidans*. *IDCases*.

[17] Taylor P., Fischbein L. (1992). Prosthetic knee infection due to *Achromobacter xylosoxidans*. *The Journal of Rheumatology*.

[18] Gupta V., Nirkhiwale S., Gupta P., Phatak S. (2012). *Achromobacter xylosoxidans* mesh related infection: a case of delayed diagnosis and management. *Journal of Infection*.

[19] Eshwara V. K., Mukhopadhyay C., Mohan S., Prakash R., Pai G. (2011). Two unique presentations of *Achromobacter xylosoxidans* infections in clinical settings. *The Journal of Infection in Developing Countries*.

[20] Ahmed M. S., Nistal C., Jayan R., Kuduvalli M., Anijeet H. K. (2009). *Achromobacter xylosoxidans*, an emerging pathogen in catheter-related infection in dialysis population causing prosthetic valve endocarditis: a case report and review of literature. *Clinical Nephrology*.

[21] Aisenberg G., Rolston K. V., Safdar A. (2004). Bacteremia caused by *Achromobacter* and *Alcaligenes* species in 46 patients with cancer (1989–2003). *Cancer*.

[22] Amoureux L., Bador J., Siebor E., Taillefumier N., Fanton A., Neuwirth C. (2013). Epidemiology and resistance of *Achromobacter xylosoxidans* from cystic fibrosis patients in Dijon, Burgundy: first French data. *Journal of Cystic Fibrosis*.

[23] Manfredi R., Nanetti A., Ferri M., Chiodo F. (1997). Bacteremia and respiratory involvement by *Alcaligenes xylosoxidans* in patients infected with the human immunodeficiency virus. *European Journal of Clinical Microbiology and Infectious Diseases*.

[24] Claassen S. L., Reese J. M., Mysliwiec V., Mahlen S. D. (2011). *Achromobacter xylosoxidans* infection presenting as a pulmonary nodule mimicking cancer. *Journal of Clinical Microbiology*.

[25] Abbott I. J., Peleg A. Y. (2015). *Stenotrophomonas Achromobacter*, and nonmelioid *Burkholderia* species: antimicrobial resistance and therapeutic strategies. *Seminars in Respiratory and Critical Care Medicine*.

[26] Spilker T., Vandamme P., Lipuma J. J. (2013). Identification and distribution of *Achromobacter* species in cystic fibrosis. *Journal of Cystic Fibrosis*.

[27] Spilker T., Vandamme P., LiPuma J. J. (2012). A multilocus sequence typing scheme impliespopulation structure and reveals several putative novel *Achromobacter* species. *Journal of Clinical Microbiology*.

[28] Vandamme P. A., Peeters C., Inganäs E. (2016). Taxonomic dissection of *Achromobacter* denitrificans Coenye et al. 2003 and proposal of *Achromobacter* agilis sp. nov., nom. rev., *Achromobacter* pestifer sp. nov., nom. rev., *Achromobacter* kerstersii sp. nov. and *Achromobacter* deleyi sp. nov. *International Journal of Systematic and Evolutionary Microbiology*.

[29] Amoureux L., Bador J., Verrier T., Mjahed H., De Curraize C., Neuwirth C. (2016). *Achromobacter xylosoxidans* is the predominant *Achromobacter* species isolated from diverse non-respiratory samples. *Epidemiology Infection*.

[30] Vandamme P., Moore E. R. B., Cnockaert M. (2013). *Achromobacter* animicus sp. nov., *Achromobacter *mucicolens sp. nov., *Achromobacter *pulmonis sp. nov. and *Achromobacter *spiritinus sp. nov., from human clinical samples. *Systematic and Applied Microbiology*.

[31] Vandamme P., Moore E. R. B., Cnockaert M. (2013). Classification of *Achromobacter *genogroups 2, 5, 7 and 14 as *Achromobacter *insuavis sp.nov., *Achromobacter* aegrifaciens sp. nov., *Achromobacter* anxifer sp. nov. and *Achromobacter *dolens sp. nov., respectively. *Systematic and Applied Microbiology*.

[32] Almuzara M., Limansky A., Ballerini V., Galanternik L., Famiglietti A., Vay C. (2010). *In vitro* susceptibility of *Achromobacter* spp. isolates: comparison of disk diffusion, Etest and agar dilution methods. *International Journal of Antimicrobial Agents*.

[33] Bador J., Amoureux L., Duez J. M. (2011). First description of an RND-type multidrug efflux pump in *Achromobacter xylosoxidans, *AxyABM. *Antimicrobial Agents and Chemotherapy*.

[34] Bador J., Amoureux L., Blanc E., Neuwirth C. (2013). Innate aminoglycoside resistance of *Achromobacter xylosoxidans* is due to AxyXY-OprZ, an RND-type multidrug efflux pump. *Antimicrobial Agents and Chemotherapy*.

[35] Bador J., Neuwirth C., Liszczynski P. (2015). Distribution of innate efflux-mediated aminoglycoside resistance among different *Achromobacter* species. *New Microbes New Infections*.

[36] Iyobe S., Kusadokoro H., Takahashi A. (2002). Detection of a variant metallo-beta-lactamase, IMP-10, from two unrelated strains of *Pseudomonas aeruginosa* and an *Alcaligenes xylosoxidans* strain. * Antimicrobial Agents and Chemotherapy*.

[37] Riccio M. L., Pallecchi L., Fontana R., Rossolini G. M. (2001). In70 of plasmid pAX22, a bla(VIM-1)-containing integron carrying a new aminoglycoside phosphotransferase gene cassette. *Antimicrobial Agents and Chemotherapy*.

[38] Shin K. S., Han K., Lee J. (2005). Imipenem-resistant *Achromobacter xylosoxidans* carrying blaVIM-2-containing class 1 integron. *Diagnostic Microbiology and Infectious Disease*.

[39] Neuwirth C., Freby C., Ogier-Desserrey A. (2006). VEB-1 in *Achromobacter xylosoxidans* from cystic fibrosis patient France. *Emerging Infectious Diseases*.

[40] El Salabi A., Borra P. S., Toleman M. A., Samuelsen Ø., Walsh T. R. (2012). Genetic and biochemical characterization of a novel metallo-*β*-lactamase, TMB-1, from an *Achromobacter xylosoxidans* strain isolated in Tripoli, Libya. *Antimicrobial Agents and Chemotherapy*.

[41] Shah R. D., Tariq N., Shanley C., Robbins J., Janczyk R. (2009). Peritonitis from peg tube insertion in surgical intensive care unit patients: identification of risk factors and clinical outcomes. *Surgical Endoscopy*.

[42] Luman W., Kwek K. R., Loi K. L., Chiam M. A., Cheung W. K., Ng H. S. (2001). Percutaneous endoscopic gastrostomy–indications and outcome of our experience at the Singapore general hospital. *Singapore Medical Journal*.

[43] Li P. K., Szeto C. C., Piraino B. (2016). ISPD peritonitis recommendations: 2016 update on prevention and treatment. *Peritoneal Dialysis International*.

[44] Löser C., Aschl G., Hébuterne X. (2005). ESPEN guidelines on artificial enteral nutrition–percutaneous endoscopic gastrostomy (PEG). *Clinical Nutrition*.

[45] Krishna S., Singh S., Dinesh K. R., Kp R., Siyad I., Karim S. (2015). Percutaneous endoscopic gastrostomy (PEG) site infections: a clinical and microbiological study from university teaching hospital, India. *Journal of Infection Prevention*.

[46] Chaudhary K. A., Smith O. J., Cuddy P. G., Clarkston W. K. (2002). PEG site infections: the emergence of methicillin resistant *Staphylococcus aureus* as a major pathogen. *The American Journal of Gastroenterology*.

[47] Rao G. G., Osman M., Johnson L., Ramsey D., Jones S., Fidler H. (2004). Prevention of percutaneous endoscopic gastrostomy site infections caused by methicillin-resistant *Staphylococcus aureus*. *Journal of Hospital Infection*.

[48] Abuksis G., Mor M., Segal N. (2000). Percutaneous endoscopic gastrostomy: high mortality rates in hospitalized patients. *The American Journal of Gastroenterology*.

[49] Melgosa M., Espinazo O., Alonso A., García Perea A., Navarro M. (2004). Dialysis-associated *Alcaligenes xylosoxidans* peritonitis: a pediatric case. *Peritoneal Dialysis International*.

[50] Koçak G., Azak A., Huddam B., Yanik S., Duranay M. (2012). Continuous ambulatory peritoneal dialysis-related peritonitis in an uremic outpatient: *Achromobacter xylosoxidans*. *Blood Purification*.

[51] Cankaya E., Keles M., Gulcan E., Uyanik A., Uyanik H. (2014). A rare cause of peritoneal dialysis-related peritonitis: *Achromobacter* denitrificans. *Peritoneal Dialysis International*.

[52] Donderski R., Grajewska M., Mikucka A., Sulikowska B., Gospodarek-Komkowska E., Manitius J. (2018). *Achromobacter xylosoxidans* relapsing peritonitis and *Streptococcus suis* peritonitis in peritoneal dialysis patients: a report of two cases. *Case Reports in Nephrology*.

[53] Morrison Jr A. J., Boyce K. (1986). 4th Peritonitis caused by *Alcaligenes* denitrificans subsp. *xylosoxydans*: case report and review of the literature. *Journal of Clinical Microbiology*.

[54] Haqqie S. S., Roth M., Bailie G. R. (1995). Unsuccessful treatment of CAPD peritonitis caused by *Alcaligenes xylosoxidans* subsp. denitrificans. *Renal Failure*.

[55] El-Shahawy M. A., Kim D., Gadallah M. F. (1998). Peritoneal dialysis-associated peritonitis caused by *Alcaligenes xylosoxidans*. *American Journal of Nephrology*.

[56] Tang S., Cheng C. C., Tse K. C. (2001). CAPD-associated peritonitis caused by *Alcaligenes xylosoxidans* sp. *xylosoxidans*. *American Journal of Nephrology*.

[57] Ledger S. G., Cordy P. (2007). Successful treatment of *Alcaligenes xylosoxidans* in automated peritoneal dialysis-related peritonitis. *Peritoneal Dialysis International*.

[58] Tsai S. F., Shu K. H. (2010). CAPD peritonitis caused by *Alcaligenes xylosoxidans* in a diabetic cirrhosis patient. *Renal Failure*.

[59] Oh T. G., Baek J. H., Jeong S. J. (2011). Continuous ambulatory peritoneal dialysis-associated peritonitis caused by *Achromobacter xylosoxidans*: a case report and comprehensive literature review. *Infection Chemotherapy*.

[60] Tsai M. T., Yang W. C., Lin C. C. (2012). Continuous ambulatory peritoneal dialysis-related exit-site infections caused by *Achromobacter* denitrificans and *A. xylosoxidans*. *Peritoneal Dialysis International*.

[61] Pereira R. H., Leão R. S., Carvalho-Assef A. P. (2017). Patterns of virulence factor expression and antimicrobial resistance in *Achromobacter xylosoxidans* and *Achromobacter* ruhlandii isolates from patients with cystic fibrosis. *Epidemiology Infection*.

[62] Tom S. K., Yau Y. C., Beaudoin T., LiPuma J. J., Waters V. (2015). Effect of high-dose antimicrobials on biofilm growth of *Achromobacter* species isolated from cystic fibrosis patients. *Antimicrobial Agents and Chemotherapy*.

[63] Jarvis E. M., Hawley C. M., McDonald S. P. Predictors, treatment, and outcomes of nonPseudomonas Gram-negative peritonitis. *Kidney International*.

[64] Valdés-Sotomayor J., Cirugeda A., Bajo M. A. (2003). Increased severity of *Escherichia coli* peritonitis in peritoneal dialysis patients independent of changes in in vitro antimicrobial susceptibility testing. *Peritoneal Dialysis International*.

[65] Prasad N., Gupta A., Sharma R. K., Prasad K. N., Gulati S., Sharma A. P. (2003). Outcome of Gram-positive and Gram-negative peritonitis in patients on continuous ambulatory peritoneal dialysis: a single-center experience. *Peritoneal Dialysis International*.

[66] Szeto C. C., Chow V. C., Chow K. M. (2006). Enterobacteriaceae peritonitis complicating peritoneal dialysis: a review of 210 consecutive cases. *Kidney International*.

[67] Zurowska A., Feneberg R., Warady B. A. (2008). Gram-negative peritonitis in children undergoing long-term peritoneal dialysis. *American Journal of Kidney Diseases*.

[68] Galat S. A., Gerig K. D., Porter J. A., Slezak F. A. (1990). Management of premature removal of the percutaneous gastrostomy. *The American Surgeon*.

[69] Al-Abboodi Y., Ridha A., Fasullo M., Naguib T. H. (2017). Risks of PEG tube placement in patients with cirrhosis-associated ascites. *Clinical and Experimental Gastroenterology*.

[70] Taheri M. R., Singh H., Duerksen D. R. (2011). Peritonitis after gastrostomy tube replacement: a case series and review of literature. *Journal of Parenteral and Enteral Nutrition*.

[71] Allison M. C., Sandoe J. A., Tighe R., Simpson I. A., Hall R. J., Elliott T. S. (2009). Antibiotic prophylaxis in gastrointestinal endoscopy. *Gut*.

[72] Boullata J. I., Carrera A. L., Harvey L. (2017). ASPEN Safe practices for enteral nutrition therapy. *Journal of Parenteral and Enteral Nutrition*.

